# Safety and clinical impact of FEES – results of the FEES-registry

**DOI:** 10.1186/s42466-019-0021-5

**Published:** 2019-04-26

**Authors:** Rainer Dziewas, Matthias auf dem Brinke, Ulrich Birkmann, Götz Bräuer, Kolja Busch, Franziska Cerra, Renate Damm-Lunau, Juliane Dunkel, Amelie Fellgiebel, Elisabeth Garms, Jörg Glahn, Sandra Hagen, Sophie Held, Christine Helfer, Mirko Hiller, Christina Horn-Schenk, Christoph Kley, Nikolaus Lange, Sriramya Lapa, Christian Ledl, Beate Lindner-Pfleghar, Marion Mertl-Rötzer, Madeleine Müller, Hermann Neugebauer, Duygu Özsucu, Michael Ohms, Markus Perniß, Waltraud Pfeilschifter, Tanja Plass, Christian Roth, Robin Roukens, Tobias Schmidt-Wilcke, Beate Schumann, Julia Schwarze, Kathi Schweikert, Holger Stege, Dirk Theuerkauf, Randall S. Thomas, Ulrich Vahle, Nancy Voigt, Hermann Weber, Cornelius J. Werner, Rainer Wirth, Ingo Wittich, Hartwig Woldag, Tobias Warnecke

**Affiliations:** 1 Klinik für Neurologie, Universitätsklinikum Münster, Albert-Schweitzer-Campus 1A, 48149 Münster, Germany; 2 Asklepios Fachklinik Fürstenhof, Brunnenallee 39, 34537 Bad Wildungen, Germany; 3 GFO Kliniken Troisdorf, Betriebsstätte St. Johannes Sieglar, Wilhelm-Busch-Str. 9, 53844 Troisdorf, Germany; 4 Helios Klinikum Aue, Gartenstr. 6, 08280 Aue, Germany; 5 Klinikum Westmünsterland St.Marien-Hospital GmbH, Am Boltenhof 7, 46325 Borken, Germany; 6 Universitätsklinik für Neurologie und Neurogeriatrie, Johannes Wesling Klinkum, Universitätsklinikum der Ruhr-Universität Bochum, Hans-Nolte-Straße 1, 32429 Minden, Germany; 7 August-Bier-Klinik, Diekseepromenade 7-11, 23714 Bad Malente-Gremsmühlen, Germany; 8 Klinik für Neurologie, Klinikum Kassel, Mönchebergstraße 41-43, 34125 Kassel, Germany; 9 Neurologische Klinik Westend, Michael Wicker GmbH & Co.OHG, Dr.-Born-Straße 9, 34537 Bad Wildungen, Germany; 10 Asklepios Kliniken Schildautal, Karl-Herold-Str. 1, 38723 Seesen, Germany; 11 Benedictus Krankenhaus Feldafing GmbH & Co. KG, Dr.-Appelhans-Weg 6, 82340 Feldafing, Germany; 12 Klinik für Hals-, Nasen-, Ohrenheilkunde, Kopf- und Halschirurgie und Klinik für Neurologie, Vivantes Klinikum Neukölln, Rudower Str.48, 12351 Berlin, Germany; 13 Das Dysphagiezentrum, Dysphagienetzwerk Südsachsen, Scherbank 18, 09456 Annaberg Buchholz, Germany; 14 Klinik für Geriatrie, Marienkrankenhaus Schwerte, Schützenstr. 9, 58239 Schwerte, Germany; 15 Sozialstiftung Bamberg, Buger Straße 80, 96049 Bamberg, Germany; 16 Klinik für Neurologie, Klinikum der Goethe-Universität, Schleusenweg 2-16, 60528 Frankfurt am Main, Germany; 17 Schön Klinik Bad Aibling, Kolbermoorer Str. 72, 83043 Bad Aibling, Germany; 18 RKU, Universitäts- und Rehabilitationskliniken Ulm, Oberer Eselsberg 45, 89081 Ulm, Germany; 19 REHAB Basel, Klinik für Neurorehabilitation und Paraplegiologie, Im Burgfelderhof 40, 4012 Basel, Switzerland; 20 Klinik für Neurologie mit klinischer Neurophysiologie, Herz-Jesu-Krankenhaus Hiltrup, Westfalenstr. 109, 48165 Münster, Germany; 21 OGD Ostprignitz-Ruppiner-Gesundheitsdienste GmbH, Fehrbelliner Str. 38, 16816 Neuruppin, Germany; 22 St. Mauritius Therapieklinik Meerbusch, Strümper Straße 111, 40670 Meerbusch, Germany; 23 Mauritius Therapieklinik Meerbusch, Strümper Straße 111, 40670 Meerbusch, Germany; 24 Institute of Clinical Neuroscience and Medical Psychology, Universitätsklinikum Düsseldorf, Düsseldorf, Germany; 25 Klinik für Neurologie, Uniklinik RWTH Aachen, Pauwelsstraße 30, 52074 Aachen, Germany; 26 Klinik für Geriatrie, Ruppiner Kliniken GmbH, Fehrbelliner Straße 38, 16816 Neuruppin, Germany; 27 Neurologisches Rehabilitationszentrum Leipzig, 04828 Bennewitz, Muldentalweg 1, Germany; 28 Sektion Interdisziplinäre Geriatrie, Klinik für Neurologie, Uniklinik RWTH Aachen, Pauwelsstraße 30, 52074 Aachen, Germany; 29 Klinik für Altersmedizin und Frührehabilitation, Marien Hospital Herne, Universitätsklinikum der Ruhr-Universität Bochum, Katholische Kliniken Rhein-Ruhr, Hölkeskampring 40, 44625 Herne, Germany

## Abstract

**Background:**

At present, the flexible endoscopic evaluation of swallowing (FEES) is one of the most commonly used methods for the objective assessment of swallowing. This multicenter trial prospectively collected data on the safety of FEES and also assessed the impact of this procedure on clinical dysphagia management.

**Methods:**

Patients were recruited in 23 hospitals in Germany and Switzerland from September 2014 to May 2017. Patient characteristics, professional affiliation of the FEES examiners (physicians or speech and language therapists), side-effects and cardiorespiratory parameters, severity of dysphagia and clinical consequences of FEES were documented.

**Results:**

2401 patients, mean age 69.8 (14.6) years, 42.3% women, were included in the FEES-registry. The most common main diagnosis was stroke (61%), followed by Parkinson’s disease (6.5%). FEES was well tolerated by patients. Complications were reported in 2% of examinations, were all self-limited and resolved without sequelae and showed no correlation to the endoscopist’s previous experience. In more than 50% of investigations FEES led to changes of feeding strategies, in the majority of cases an upgrade of oral diet was possible.

**Discussion:**

This study confirmed that FEES, even when performed by less experienced clinicians is a safe and well tolerated procedure and significantly impacts on the patients’ clinical course. Implementation of a FEES-service in different clinical settings may improve dysphagia care.

**Trial registration:**

ClinicalTrials.gov NCT03037762, registered January 31st 2017.

## Introduction

Neurogenic dysphagia is one of the most frequent and life-threatening symptoms of neurological disorders such as stroke, traumatic brain injury, Parkinson’s disease, dementia, multiple sclerosis, and different neuromuscular disorders [1–7]. In view of the demographic shift, especially with increasing numbers of very old people, these already alarming figures will further increase in the future since many underlying pathologies are age related. The clinical consequences of dysphagia are serious and, in general, directly linked to the patient’s overall prognosis. Irrespective of the underlying disease the set of typical complications comprises aspiration pneumonia, malnutrition and dehydration ultimately leading to an increase in mortality [8]. Apart from these medical issues, dysphagia has a significant impact on the psychological well-being of affected individuals and has been linked to social isolation, low mood and depression [9, 10].

Since the first description of Flexible Endoscopic Evaluation of Swallowing (FEES) was published in 1988 by Langmore and co-workers [11], this particular technique has turned into one of the most commonly used methods for the objective assessment of swallowing worldwide [12]. In terms of day-to-day practicality, the merits of FEES are that (i) it can be performed at the bedside, thus facilitating examination of severely motor-impaired, bedridden or uncooperative patients, for example in the intensive care unit or the stroke unit; (ii) follow-up examinations can be performed at short notice and, if necessary, frequently; and (iii) oropharyngeal secretion management and efficacy of clearing mechanisms, such as coughing and throat clearing, can be assessed simply and directly. In several studies FEES has been successfully applied in a wide range of specific disorders, such as stroke [13], traumatic brain injury [14], cerebral palsy [15], Parkinson’s disease and atypical Parkinsonian syndromes [16, 17], different types of dementia [4], amyotrophic lateral sclerosis [18], Kennedy’s disease [19], and head and neck cancer [20]. In addition, FEES is also being increasingly used in paediatrics [21], geriatrics [22] and intensive-care medicine [23, 24]. The growing interest in this technique is also reflected by the development of systematic educational curricula put forward by different medical societies. Remarkably, these curricula are not confined to a specific medical profession but are all designed as interdisciplinary concepts involving a variety of healthcare professionals being engaged in the management of dysphagia [25–27].

In spite of the increasing dissemination of FEES, there are only few studies that evaluate procedure related side effects and the clinical benefits related to providing this tool for objective dysphagia evaluation. This multicenter trial, the FEES-registry, therefore prospectively collected data on the safety of FEES and also assessed the impact this procedure had on dysphagia management in the studied patient cohort.

## Patients and methods

Patients were prospectively recruited in 23 hospitals in Germany and Switzerland from September 2014 to May 2017. Trial sites were identified among those hospitals actively supporting the German FEES education initiative. Trial sites included 10 neurological departments, 9 rehabilitation facilities and 4 geriatric departments. Patients were considered eligible for this study if a FEES was scheduled during their treatment either within the in- or outpatient service. There were no in- or exclusion criteria with regards to the patients’ main diagnosis or treatment facility. The study protocol was approved by all involved ethics committees, and all patients or their legal representative provided written informed consent. The FEES-registry was registered as NCT03037762.

### Patient characteristics

The following epidemiological and clinical variables were recorded: sex and age, main diagnosis, Barthel index [28] and the use of antithrombotics, antiplatelets or anticoagulation. Directly prior to FEES the Richmond Agitation and Sedation Scale (RASS) was scored [29]. In addition, using a previously established definition of so called “complex patients”, it was noted, whether the examination was particularly challenging, which was considered to be the case if patients showed a respiratory impairment (increased respiratory rate, need for oxygen supply), were restless (due to for example a movement disorder), had a limited understanding of the situation or a fluctuating vigilance, or had a tracheal cannula in place [25].

### Professional affiliation of the examiner

The profession of the involved examiners was documented (either physician or speech-and-language therapist (SLT)). Their previous experience in performing FEES was categorized in < 30 FEES, 30–200 FEES, 201–500 FEES, or > 500 FEES.

### Cardiorespiratory monitoring and side-effects

Where possible, heart rate and oxygen saturation were monitored during FEES and the following four values were noted: i) pretest, ii) highest value during FEES, iii) lowest value during FEES, iv) posttest. Blood pressure was measured twice, immediately prior and directly after FEES. Apart from that the following side-effects were noted: Epistaxis, laryngospasm, bradycardia, decrease of the level of consciousness (i.e. for example from alert to somnolent). After completion of the examination, patients were asked to rate the level of discomfort associated with FEES as “none”, “mild”, “moderate”, or “severe”.

### Rating of feeding strategy and dysphagia

Prior to FEES the oral intake of patients was rated with the Functional Oral Intake Scale (FOIS) [30], which ranges from 1 (no oral intake) to 7 (total oral intake with no restrictions). Based on the FEES results, severity of swallowing dysfunction was classified according to a 4-grade dysphagia severity scale that has previously been developed and published [16, 31] (0 = no relevant dysphagia, 1 = mild dysphagia (premature spillage and/or residues, but no penetration/aspiration events), 2 = moderate dysphagia (penetration/aspiration events with one consistency), 3 = severe dysphagia (penetration/aspiration events with two or more consistencies)). In addition, based on the FEES findings and the global clinical situation a new FOIS score was defined with the difference between the FOIS-scores pre- and post-FEES reflecting the clinical impact of this examination. In addition, it was noted whether in patients with a tracheal cannula in place decannulation was recommended after FEES.

### Statistical analysis

Statistical analyses were carried out with SPSS 25.0 for WINDOWS (SPSS Inc). The paired-samples t-test was used to compare pre- and post-test blood pressure, the repeated-measures ANOVA was used to compare oxygen saturation and heart rate prior, during and after FEES. Categorical data were analyzed using the chi-square test. For correlation analysis the Pearson-correlation coefficient was calculated.

## Results

As summarized in Tables 1, 2401 patients were included in the FEES-registry. Mean age was 70 years and 42.2% were female. Mean RASS score was close to 0 and mean Barthel index was 35. Close to 19% of patients were on anticoagulation, about one third of patients received antiplatelets and more than 40% were treated with antithrombotic drugs. More than 45% of patients were rated as complex cases, most frequently cited conditions were disorientation (20.7%), presence of a tracheal cannula (18.6%), and fluctuating consciousness (16.2%). The most common main diagnosis of patients enrolled in this study was stroke (61%), followed by Parkinson’s disease (6.5%), CIP (5.6%), Motor-neuron disorders (3.1%) and dementia (2.7%). Non-neurological diseases were rare and constituted malignoma (2.0%), psychogenic dysphagia (1.4%), cervical spine surgery (0.8%), pneumonia (0.5%) and esophageal diseases (0.5%).

**Table 1:** Epidemiological and clinical characteristics of the patient cohort. Numbers in brackets give the number of patients with complete datasets with regards to the specific items

General characteristics (*N* = 2236)	
Age	69.8 (14.6)
Female gender	1013 (42.2)
Barthel	35 (35.4))
RASS	−0.1 (0.81)
Anticoagulation	451 (18.8)
Anti-platelets	796 (33.2)
Antithrombotic drugs	1005 (41.9)
Specific characteristics (*N* = 2330)	
Complex patients	1089 (45.4)
Respiratory problems	279 (11.6)
Tracheal cannula	447 (18.6)
Agitation	161 (6.7)
Disorientation	496 (20.7)
Fluctuating vigilance	390 (16.2)
Main Diagnosis (*N* = 2401)	
Stroke	1465 (61.0)
Stroke with Thrombolysis	393 (26.8)
Parkinson’s Disease	157 (6.5)
Critical-Illness Polyneuropathy	135 (5.6)
Motorneuron Disorder	75 (3.1)
Dementia	64 (2.7)
Malignoma	48 (2.0)
Movenent Disorders (other)	41 (1.7)
Enzephalopathia	37 (1.5)
Traumatic Brain Injury	36 (1.5)
Meningitis/Enzephalitis	36 (1.5)
Myasthenia gravis	35 (1.5)
Immune-mediated neuropathy	34 (1.4)
Psychogenic dysphagia	34 (1.4)
Seizure	33 (1.4)
Myopathy	29 (1.2)
Cervical spine surgery	20 (0.8)
Multiple Sclerosis	18 (0.7)
Pneumonia	13 (0.5)
Esophageal diseases	12 (0.5)
Other/Missing	79 (3.3)

Most of the examinations were done in an acute care facility (70.5%), 20.5% of patients were enrolled in rehabilitation clinics and 9.0% were seen as outpatients (Table 2). Inpatients were examined at all levels of care, i.e. normal wards (46.6%), intermediate care units (31.1%) and intensive care units (22.4%) (Table 2). In nearly all FEES SLTs were involved (95.5%), 41.2% were done by a team of SLTs without involvement of other personnel, physicians took part in 58.8% of examinations. The majority of FEES was done by a highly experienced clinician; however, in 17.7% of cases the endoscopist had done less than 30 FEES before (Table 2). The mean examination time devoted to the endoscopic procedure was close to 10 min. This figure does not include the additional time needed for preparation of FEES, for writing the report, for communicating the findings within the treating team and for the cleaning procedure.

**Table 2:** Features of the clinical context, in which FEES was carried out

Setting (N = 2401)	
Outpatient service	216 (9.0)
Acute care facility	1692 (70.5)
Rehabilitation facility	493 (20.5)
Level of care (for inpatients, *N* = 1735)	
Normal ward	808 (46.6)
Intermediate care unit	539 (31.1)
Intensive care unit	388 (22.4)
Examiner’s profession (*N* = 2389)	
Physician involved	1404 (58.8)
SLT involved	2282 (95.5)
SLT alone	985 (41.2)
Examiner’s experience (N = 2401)	
< 30 FEES	420 (17.7)
30–200 FEES	609 (25.6)
201–500	389 (16.4)
> 500	960 (40.4)
Examination time (min.) (*N* = 2362)	9.84 (5.89)

**Fig. 1:**
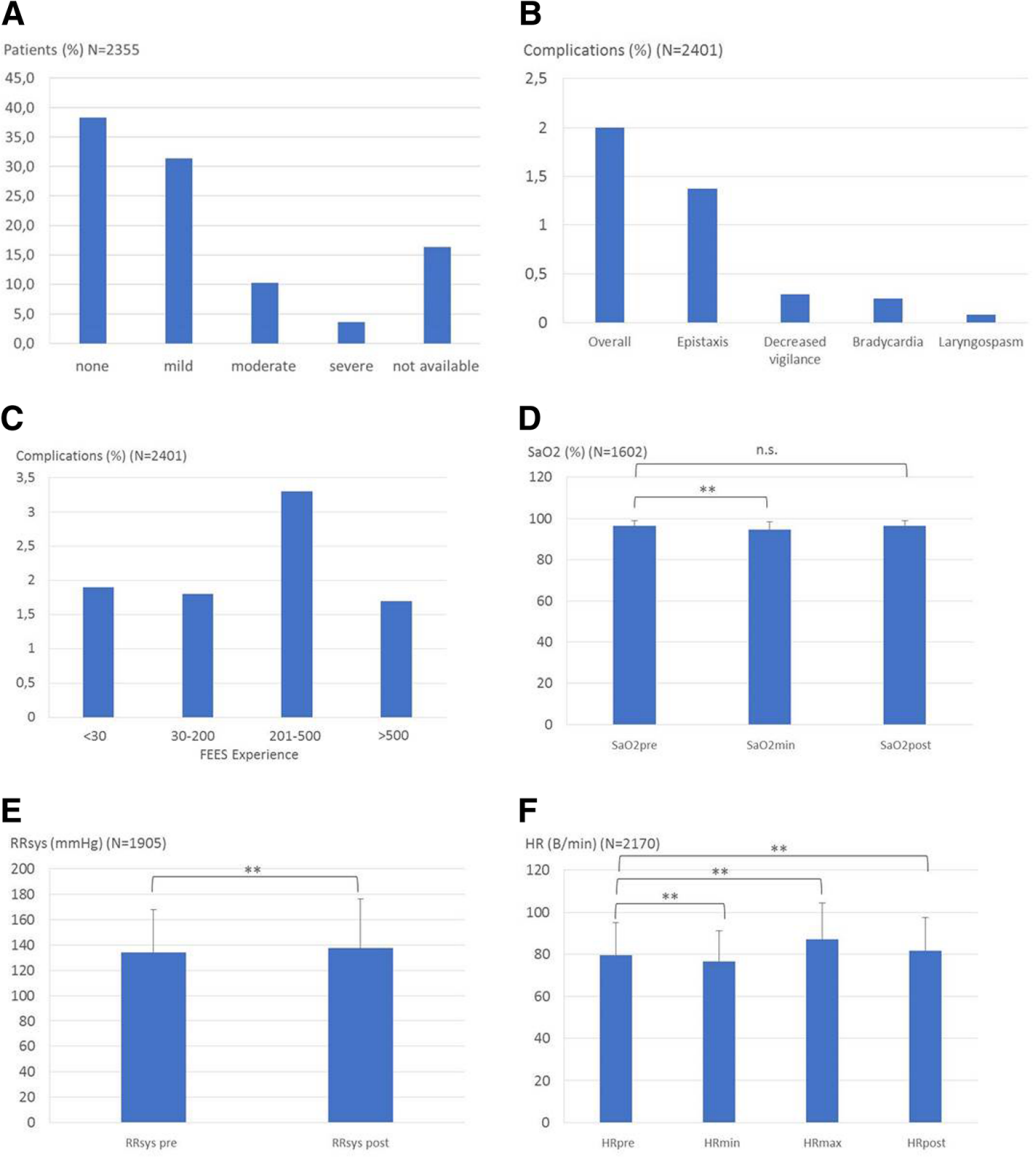
Tolerance, complications and alterations of cardiorespiratory parameters during FEES. **a**: Patients’ rating of FEES-associated discomfort ranging from none to severe; **b**: Incidence of complications; **c**: Incidence of complications in relation to the clinician’s FEES-experience. Numbers below columns give the number of FEES performed during prior training; **d**: Procedure-related changes of oxygen saturation (SaO2); E: Procedure-related changes of systolic blood pressure (RRsys); F: Procedure-related changes of heart rate (HR)

FEES was tolerated well by the patients with nearly 70% rating the procedure as not uncomfortable or mildly uncomfortable. 10.2% stated that FEES was moderately uncomfortable and 3.7% experienced severe discomfort with the remaining 16.3% not being able to provide a rating due to their underlying illness (Fig. 1A).

Complications were reported in 2% of examinations (Fig. 1B). In 33 cases (1.37%) epistaxis occurred, a decreased consciousness was noted in 7 patients (0.29%), 6 patients (0,25%) developed bradycardia and in 2 patients (0.08%) a laryngospasm was reported. All of these complications were self-limited and terminated within a few minutes without specific intervention. The incidence of complications was not related to the endoscopist’s experience. In fact, FEES done by endoscopists with a professional experience of 200–500 examinations featured the highest rate of complications, although without significant differences between groups (Fig. 1C). As shown in Fig. 1 D-F FEES was associated with significant changes in cardiorespiratory parameters. Thus, oxygen saturation dropped in mean by 1.8%, systolic blood pressure increased by 3.5 mmHg, and maximum heart rate increased by 7.4 bpm and minimum heart rate decreased by 3.2 bpm. The clinical impact of these alterations was however limited. Thus, post-intervention oxygen saturation and heart rate had nearly returned to the respective baseline-values and no associated complications were observed.

**Fig. 2:**
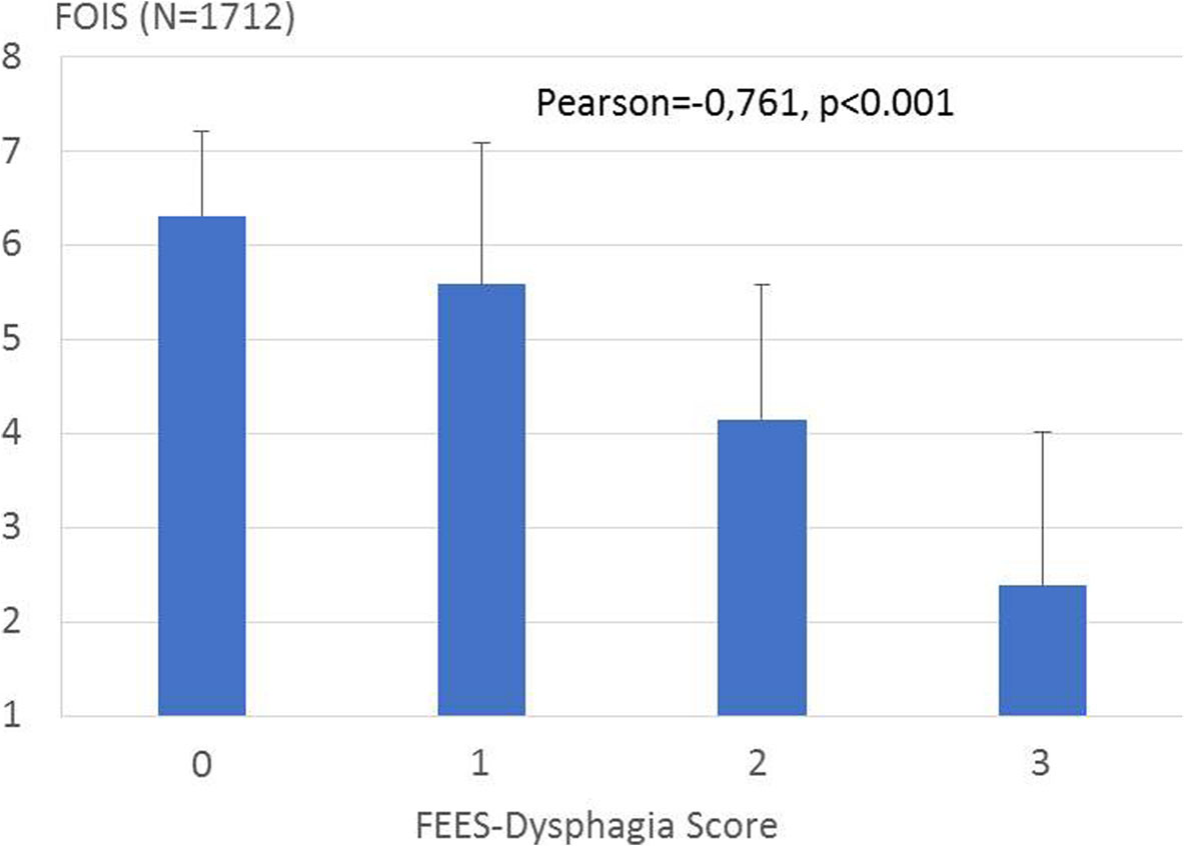
Correlation of the FEES-based dysphagia score with the Functional Oral Intake Score (FOIS)

The 4-grade FEES-based dysphagia score correlated well with the FOIS score (Pearson correlation coefficient − 0.761, *p* < 0.001; Fig. 2). A dysphagia score of 0 corresponded to a FOIS score between 6 and 7, a dysphagia score of 1 to a FOIS score between 5 and 6, a dysphagia score of 2 to a FOIS score of close to 4, and a dysphagia score of 3 to a FOIS score between 2 and 3.

**Fig. 3:**
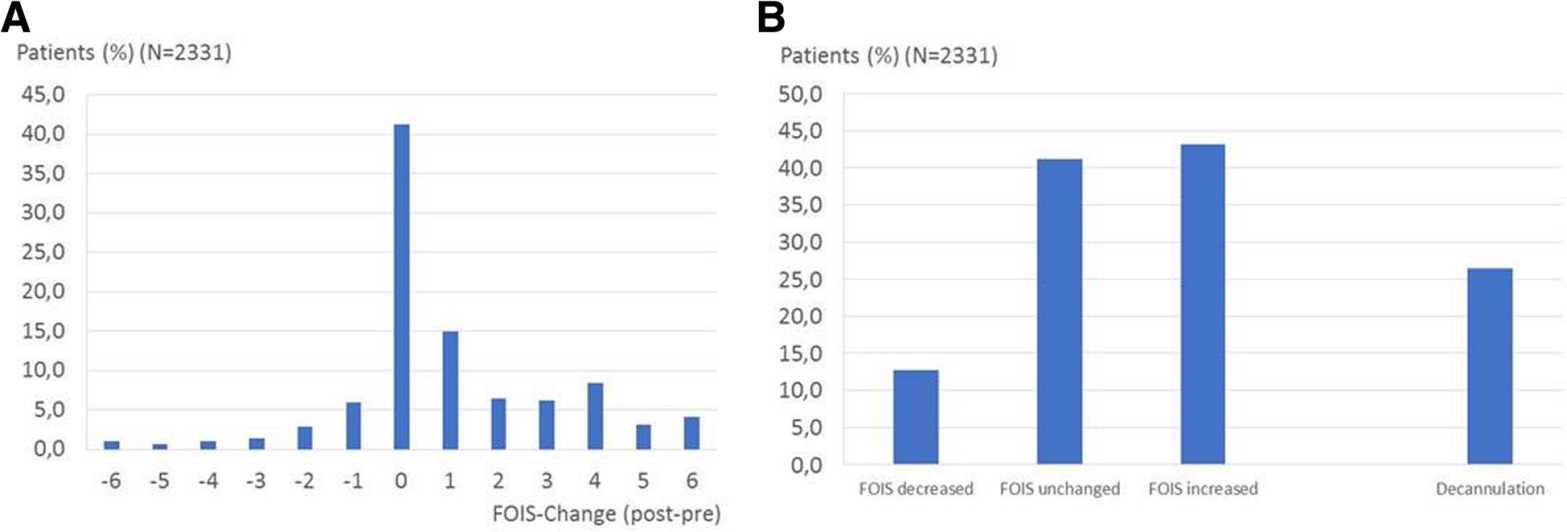
Changes of dysphagia management after FEES. **a**: Detailed changes of FOIS score after FEES. A positive value indicates an upgrade of the FOIS score after FEES, a negative value indicates more restrictive feeding strategy. **b**: Summary of FOIS changes and management of tracheotomised patients with regards to decannulation

In more than 50% of cases FEES led to changes of feeding strategies (Fig. 3). Whereas in 43.2% of patients an upgrade of the oral diet was possible and in more than 20% of patients the FOIS scale increased by 3 or more points (Fig. 3A), oral diet needed to be restricted after FEES in 12.7% of patients. In the subgroup of tracheotomized patients decannulation was possible in more than 25% of them (Fig. 3B).

## Discussion

The FEES-registry assessed the safety and clinical impact of FEES in a prospective multicenter design across different levels of care facilities in a heterogeneous patient cohort. The study’s first main finding was that the procedure was safe and well tolerated, and complications, in particular laryngospasm, epistaxis and hypotensive episodes were very rare and always self-limited, thereby corroborating reports from the literature [32–36]. Secondly, this study showed that the incidence of procedure-related side-effects was not related to the endoscopist’s experience. Therefore, FEES seems to be safe even when performed by professionals with limited prior training. This result supports recently published formalized training curricula for FEES that suggest that after taking part in a dedicated workshop, conducting 60 supervised examinations and passing a practical test physicians and SLTs can safely perform this procedure [25–27]. Third, this trial showed that FEES was associated with discernible but clinically insignificant alterations of cardiovascular parameters. Interestingly, and in line with a smaller previous trial exclusively focusing on acute stroke patients [36], the recorded mild increases of heart rate and systolic blood pressure were clearly less pronounced than encountered during placement of nasogastric tubes in acute stroke patients with dysphagia [37]. In the latter scenario a mean increase of systolic blood pressure of 35 mmHg (as opposed to 3.5 mmHg in the present trial) and a mean increase of heart rate of 23 bpm (as opposed to 3.2 bpm in the present trial) were noted. Therefore, it may be concluded that the FEES procedure, even if examination times may be longer, is not as unpleasant as any procedure involving blind manipulation within the nostrils and the pharynx such as placing nasogastric tubes or nasotracheal suctioning. Fourth, this study showed that a simple FEES-based algorithm grading dysphagia severity according to efficiency and safety of swallowing with regards to different consistencies correlates well with the less swallowing specific FOIS score. In the past, this algorithm was used in patients with movement disorders [16, 31]. However, since the present multicenter trial has demonstrated that the algorithm (i) is readily applicable in different diagnostic groups and (ii) is able to grade dysphagia in a clinically meaningful way, it may be assumed that this FEES-score could be helpful in everyday patients’ care and might be useful as an endpoint in clinical studies devoted to the topic of neurogenic dysphagia [38]. Finally, the present study also collected data with regards to the impact of FEES on dysphagia management. In more than 50% of patients FEES led to changes in the feeding strategy. Furthermore, in more than 25% of the subgroup of 447 tracheotomized patients, decannulation was deemed safe based on FEES-findings. These results corroborate existing literature, which usually focused on specific patient cohorts. Thus, in a recent study recruiting stroke patients and adopting a pre-post-design, Bax and co-workers showed that providing FEES-service on a stroke unit reduced the incidence of post-stroke pneumonia and increased the proportion of patients leaving hospital on a regular diet [39]. Hafner et al. reported clinical consequences of using FEES in a critical care setting in recently extubated patients [23]. Based on FEES prolonged non-oral feeding was required in 49.7% of patients, in 6.3% a tracheostomy was performed, an oral diet was started in 30.7% and tracheostomies were closed in 22.9%. Evaluating swallowing function in tracheostomized neurointensive care patients with a FEES-based decannulation algorithm, Warnecke et al. demonstrated that safe decannulation was possible in more than 50% of patients, whereas only about 30% of them would have been decannulated based on clinical swallowing evaluation alone [40]. Taken together, these studies provide first evidence that implementation of a FEES-service in different clinical settings may improve dysphagia care.

The strengths of this prospective observational study are its multicentre design, the inclusion of a heterogeneous patient cohort and the specific documentation of different features of the examination setting and of the respective results. However, some limitations are apparent. First, trial sites were chosen among those hospitals actively supporting the German FEES education initiative. Therefore, it is conceivable that sites with a more advanced level of proficiency were chosen against less experienced centres, which may have introduced a bias into the findings. Second, the study did not include documentation of potentially eligible patients that for various reasons were not recruited in the end. Hence, a selection bias cannot fully be ruled out. Third, probably reflecting the usual distributions of different disease categories in a given patient collective, stroke was by far the most common disease, whereas other disorders were significantly rarer. Thus, the generalizability of the study’s conclusions may be limited to a certain extent. Fourth, the documentation of how FEES was performed in detail was for reasons of practicability limited. Therefore, for example, it was not recorded whether topical anesthesia had been used, a factor that may well have been related to patients’ comfort [41] Fifth, there was no central reading of FEES findings and, sixth, for some study items the proportion of missing data was rather high. Both of these aspects may have impacted the scientific validity of the study’s results. Finally, while this study showed that FEES was safe even in the hands of less experienced endoscopists, the quality of the examinations and the derived conclusions were not scrutinized and evaluated.

In conclusion, this study confirmed that FEES, even when performed by less experienced clinicians, is a safe procedure with only moderate associated alterations of cardiovascular parameters. FEES had a significant impact on dysphagia management and by adopting a simple FEES-based dysphagia score, FEES showed to provide a clinically meaningful assessment of overall dysphagia severity.

## Abbreviations

CIP: Critical Illness Polyneuropathy
FEES: Flexible Endoscopic Evaluation of Swallowing
FOIS: Functional Oral Intake Scale
